# OA11.03. Mediators of the effects of yoga and stretching on chronic low back pain (cLBP) outcomes: results from the YES RCT

**DOI:** 10.1186/1472-6882-12-S1-O43

**Published:** 2012-06-12

**Authors:** K Sherman, R Wellman, A Cook, D Cherkin

**Affiliations:** 1 Group Health Research Institute, Seattle, USA

## Purpose

We previously reported that yoga and intensive stretching had equivalent benefits for persons with chronic back pain and both were superior to self care. As part of this trial, we explored whether physical, cognitive, affective and physiological factors mediated the effects of yoga on patient outcomes.

## Methods

228 persons with non-specific cLBP recruited from primary care and the general community were randomized to 12 weekly 75-minute classes of either yoga or intensive stretching, or to a self-care book. Back-related function (Roland-Morris Disability Scale), symptoms (0-10 score) and psychological mediators (fear avoidance, body awareness, self-efficacy, psychological distress, perceived stress, positive affect) were assessed at baseline and 6, 12, and 26 weeks later by blinded interviewers. Physical function was assessed at baseline and 12 weeks and saliva samples were collected for cortisol and DHEA analyses at baseline, 6 and 12 weeks. Open-ended questions were asked about benefits of yoga and stretching. Statistical analyses for mediators were conducted using the framework of Baron and Kenney.

## Results

95% of participants responded to at least one follow-up interview. Of the potential mediators, only self-efficacy decreased significantly from baseline to 6 weeks for both interventions (p=0.0015 and 0.0129). Cortisol awakening response was marginally significant for yoga (p=0.08). For yoga, 18% of the effect was mediated by increased self-efficacy, 8% by cortisol awakening response, and 21% by any of the mediators. For exercise, 8% of stretching was mediated by self-efficacy. In response to open-ended questions about benefits, >20% of participants mentioned: learning new exercises (both groups); relaxation, increased awareness and the benefits of breathing (yoga), and benefits of regular practice (stretching).

## Conclusion

While both interventions were superior to self-care, our mediator analysis showed these benefits were not well explained by our measured “mediators”. Qualitative data suggest that yoga and stretching may exert comparable benefits through partially distinct mechanisms.

